# Searching the internet for health information about bipolar disorder: some cautionary issues

**DOI:** 10.1186/2194-7511-1-22

**Published:** 2013-10-17

**Authors:** Scott Monteith, Tasha Glenn, Michael Bauer

**Affiliations:** Michigan State University College of Human Medicine, Traverse City Campus, 1400 Medical Campus Drive, Traverse City, MI 49684 USA; ChronoRecord Association, Inc., Fullerton, CA 92834 USA; Department of Psychiatry and Psychotherapy, Universitätsklinikum Carl Gustav Carus, Medical Faculty, Technische Universität Dresden, Dresden, 01307 Germany

**Keywords:** Bipolar disorder, Internet, Web sites

## Abstract

Adults routinely use the Internet as a source of health information. Patients with bipolar disorder and caregivers should be encouraged to increase their knowledge of this complex illness, including through the Internet. However, patients, caregivers, and physicians should be aware of potential perils when searching the Internet for health information, including loss of privacy, quality of web site content, and Internet scams. This review summarizes these cautionary issues. The digital divide remains and includes a lack of technical skills and competency in searching and appraising web sites, in addition to limited access to the Internet. Physicians should provide patients with a list of trustworthy web sites and a brief printed handout on concerns related to searching the Internet. More studies of the use of the Internet by patients with bipolar disorder are needed.

## Review

### Introduction

Most understanding of the use of the Internet as a source of health information comes from surveys of the general public. In the US, 70% to 80% of all adult Internet users seek health information online (Fox and Duggan 2013; Taylor 2010). If Internet access is available, there is no extra cost to reach a large number of health web sites. Patients can search on their own schedule, 24 hours a day and in a variety of settings. Information can be read at the patient's own pace (Korp 2006). Although most of the health information obtained on the Internet is used to supplement traditional sources such as physician office visits, more than one third have used the Internet to self-diagnose (Fox and Duggan 2013). Generally, people search for information about a specific disease or condition, with depression being the third highest ranked health search term in 2102 (Google Trends 2012). People who are more likely to search the Internet for health information are female, sicker, regular Internet users, or have a stigmatized illness (Berger et al. 2005; Rice 2006; Houston and Allison 2002). For those with a chronic illness, Internet searching is especially important since patient knowledge generally leads to improvement in self-management skills and treatment adherence (Williams et al. 1998; Andrus and Roth 2002).

It may be particularly beneficial for those with bipolar disorder to obtain information online due to the disability and poor quality of life that may follow a diagnosis (Huxley and Baldessarini 2007; Michalak et al. 2005; Bauer et al. 2009). For patients with a recent diagnosis, information learned on web sites may reinforce that bipolar disorder is a serious illness with a complex and unpredictable course, which requires specialized help from a psychiatrist and treatment with psychotropic drugs. Information found on the Internet may help the patient communicate with and understand the physician's advice, and may increase the health care utilization of those who feel stigmatized by a psychiatric diagnosis (Berger et al. 2005). Many mental health sites also contain considerable general health information and promote healthy habits, which may be beneficial (Korp 2006). Learning more about bipolar disorder on the Internet may help the patient to participate more actively in their care, and it should be welcomed and encouraged by the physician.

The use of the Internet as a source of health information is also important for caregivers for the chronically ill. Caregivers make up about 35% of online health information seekers, who frequently search for the newest treatments and medications, and typically return to the same health web sites (Cain et al. 2000). Since much of the public does not understand the symptoms of mental illness (Jorm 2000; Mojtabai et al. 2002), information on mental health web sites that is aimed at family and caregivers might be particularly useful. Indeed, a lack of knowledge of the symptoms and course of bipolar disorder may undermine the critical supportive role of caregivers (Berk et al. 2013). Family education may also facilitate early intervention for mental illnesses that arise during adolescence or young adulthood (Kelly et al. 2007), such as bipolar disorder.

The potential risks of using the Internet as a source of health information receive far less attention and discussion than the benefits. This review summarizes these cautionary issues. Although most studies of Internet use involve the general public and searching for nonspecific health information, the risks are applicable to all users, and the issues of most concern for patients with bipolar disorder will be highlighted.

### Role of search engines

Eighty percent of users looking for health information on the Internet start with a general search engine (Fox and Duggan 2013). About two thirds of users believe search engines to be an unbiased source of information, with little understanding of the business models for search engine companies (Fallows 2005). As an example, Google has about two thirds of the market share in the US (Forbes 2012). Just as broadcast television provides free programming to attract an audience to sell advertising, Google provides free searches to sell advertising on the search results pages. In the words of Google CEO Eric Schmidt, ‘We are an advertising company’ that makes about 98% of its money from advertising (Schonfeld 2009). Unlike traditional media, which shows the same programs and advertising to all, Google provides personalized search results and advertising, referred to as targeted advertising (Google 2009). Targeted advertising uses proprietary algorithms to profile individuals based on large quantities of information such as search histories, clickstreams (route navigated on web site), email connections, and physical location, and these profiles are sold to advertisers. There is no standard result when someone types in a search term into Google (Vaidhyanathan 2012). The search results and advertising in Google are tailored to provide information that will facilitate spending by the user, and not to increase knowledge (Vaidhyanathan 2012). Therefore, people typing in search terms for bipolar disorder into Google may obtain very different search results. Moreover, unlike with traditional media, 62% of users could not distinguish between paid search results (advertising) and unpaid search results (Fallows 2005). Personalization has expanded beyond search engine results, with real-time bidding on user profiles to display advertising on devices such as smartphones and tablets (Rosen 2012).

### Web site selection

More than 95% of users select a web site from the first page of search results when using a general search engine (Cornwell 2010), including health information search results (Eysenbach and Köhler 2002). Once on a health web site, users judge credibility primarily by the page content rather than by checking the author credentials such as an ‘about us’ page (Fox and Rainie 2002; Eysenbach and Köhler 2002). The look and feel of health web sites is important to users, and sites with high-quality content may be rejected because of limited visual appeal or difficulty in navigation (Sillence et al. 2007). In a study of 31 health web sites, users correlated the credibility of the information with the visual design of the site (Robins et al. 2010). Users do not like health web sites that seem overly commercial (Fox and Rainie 2002; Sillence et al. 2007) and may prefer charitable over government sites (Sillence et al. 2007). Additionally, some users prefer health web sites that tailor information to contextual variables such as age, sex, cultural or racial identity, or physical location (Marton 2003; Sillence et al. 2007). After searching, patients often cannot remember the health web site from which they obtained information (Eysenbach and Köhler 2002). In addition to information sites, about one fourth of adults read about someone else's experience with medical issues in the last month (Fox and Duggan 2013).

Although there is an unprecedented amount of information on mental illness available on the Internet, the quality of information on mental health web sites varies widely. Reviews by medical professionals of the content of mental health web sites, including those that focus on bipolar disorder and depression, are quite varied, frequently reporting that content is of poor to good quality (Barnes et al. 2009; Griffiths and Christensen 2000; Berland et al. 2001; Eysenbach et al. 2002; Morel et al. 2008). However, the quality of content about mental health also varies widely in traditional media formats such as printed pamphlets and magazines (Eysenbach et al. 2002). Many web sites require high reading levels to comprehend (Eysenbach et al. 2002; Barnes et al. 2009,2001; Gralton et al. 2010), which is of concern as some patients with bipolar disorder experience neurocognitive impairment even when euthymic (Wingo et al. 2009). Since there is no professional or governmental review and approval of the content of health information web sites, many rating instruments and markers, such as Health on the Net (HON), were developed to help discriminate among web sites (Wilson 2002). However, patients do not use these quality markers (Eysenbach and Köhler 2002; Bernstam et al. 2005), and reviewers often report limited association between markers and web site quality (Morel et al. 2008; Griffiths and Christensen 2000; Shon et al. 2000). There is limited consensus among experts on what constitutes a quality web site (Turow et al. 2003; Eysenbach et al. 2002). More research is also needed on the patient perspective of the usefulness of web sites about bipolar disorder.

Health information from social Internet sites may have a more direct impact on the decisions of some users (Weaver et al. 2009), including patients with bipolar disorder (Bauer et al. 2013b), but are used by far fewer people (Hesse et al. 2005) and are beyond the scope of this paper.

### Pharmaceutical web sites

Patients with bipolar disorder want more information about the medications they take and especially about possible side effects (Bowskill et al. 2007). As more people use the Internet as a source of health information, pharmaceutical companies are expanding direct-to-consumer (DTC) promotions onto the web (Choi and Lee 2007). FDA regulation allows broadcast advertisements to reference a web site as a source of prescribing information (FDA 2010), and pharmaceutical web sites provide more detailed information than in print advertisements (Macias and Lewis 2004). Notably, pharmaceutical company web sites for brand name psychiatric medications are usually ranked on the first page in all major search engines, as demonstrated in a search for antidepressants (Graber and Weckmann 2002). There are several areas of concern with pharmaceutical web sites. Some users may not consider pharmaceutical web sites to be advertising (Choi and Lee 2007). Most users feel that pharmaceutical web sites provide information that is comprehensive and not misleading (Wymer 2010), although sites may not provide sufficient information on drug associated risks (Davis et al. 2007; Huh and Cude 2004). In a study of all drugs with DTC expenditures of at least US$5 million between January to June 2005, only half the web sites reported all side effects occurring at ≥10% incidence (Davis et al. 2007).

Although the DTC advertising appeals are similar in web and print advertisements, web advertisements contained more than twice as many monetary incentives (Macias and Lewis 2004). Of the 90 DTC sites studied, 52% offered some type of monetary inducement including rebates, coupons, free trials, and offers for merchandise. Most of these financial inducements require site registrations, but many DTC web sites do not provide sufficient or clear privacy policies (Sheehan 2005).

### Web sites for dietary supplements

The use of dietary supplements is increasing in patients with bipolar disorder (Andreescu et al. 2008; Unützer et al. 2000; Druss and Rosenheck 2000). Dietary supplements are routinely marketed on the Internet (Morris and Avorn 2003), and these web sites have a high potential for containing misleading information. In a study of 422 web sites for 8 widely used herbal supplements, about half claimed to treat, prevent, or cure specific diseases and about half omitted the required standard federal disclaimer (Morris and Avorn 2003). Additionally, many people mistakenly believe that dietary supplements are subject to the same regulation by the FDA as over-the-counter nonprescription drugs (Morris and Avorn 2003). Patients with bipolar disorder may also be unaware of potential risks associated with contamination and dosage inconsistency of dietary supplement products (Andreescu et al. 2008; Petroczi et al. 2011), and of potential interactions with drugs prescribed for psychiatric and medical conditions (Izzo 2012; Andreescu et al. 2008; Wong et al. 1998).

### Privacy issues

Patients should be cautioned about disclosing personal information to a web site when attempting to obtain health information. The privacy policies of Internet health web sites are often difficult to read and require the equivalent of 2 years of college to comprehend (Graber et al. 2002). Some web sites ask directly for personal information, such as to join a community forum, or may require the use of cookies or other software that tracks online behavior. Some health sites sell data to a variety of entities including research organizations and commercial data brokers. In a study of 120 popular web sites including health sites, 56% sent private information to data brokers, increasing to 75% when counting the user ID (Krishnamurthy et al. 2011). A study of 20 health web sites found that 13 contained third party tracking elements (Huesch 2013). Other health sites obtain information anonymously through means such as surveys, symptom checkers, health assessments, or subscriber lists (Sheehan 2005), but even data provided anonymously may have unintended consequences when combined with other data (Narayanan and Shmatikov 2010; Ohm 2010). Powerful re-identification algorithms exist, such that any data parameter can be used for identification when combined with other data (Narayanan and Shmatikov 2010; Friedland et al. 2011). Privacy concerns of US Internet users have increased since 2002, are primarily related to disclosure of personal information to third parties, and were influenced by the publicity surrounding data breaches, identity theft, activity tracking, and personalization (Anton et al. 2010). It is not known if increased consumer awareness of privacy issues will impact the use of the Internet to obtain health information.

### Internet scams

Since most patients do not check the legitimacy of the content of health web sites (Eysenbach and Köhler 2002; Bernstam et al. 2005), searches may inadvertently end up at credible-looking store fronts for fraudulent online pharmacies. Also, about one third of unsolicited email or spam includes offers of health products, primarily medications, with links to such web sites (Gernburd and Jadad 2007). The majority of orders at rogue pharmacies originate in the US, including 85% of orders for nonlifestyle drugs such as antipsychotics (Kanich et al. 2011). Patients attempting to fill legitimate prescriptions from rogue pharmacies, presumably to save money, should be aware that any drugs delivered may be substandard or counterfeit (Kanich et al. 2011; Gernburd and Jadad 2007). Many of the links in email spam are to sites that merely collect personal information and deliver no products (Gernburd and Jadad 2007).

Considerable information is available online about illegal and recreational drugs (Nielsen and Barratt 2009; Halpern and Pope 2001), which is of concern given the high rates of substance abuse in patients with bipolar disorder (Strakowski et al. 2000). Recreational drug sites may also connect to rogue online pharmacies that allow purchase of prescription drugs without following appropriate US laws (Nielsen and Barratt 2009).

### Digital divide

It is easy to forget that digital access remains a challenge to many in the US. As of the 2010 census, 25% of US households are without Internet access (US Census 2010) and Internet adaption rates have steadied (Zickuhr and Smith 2012). Only about half of Americans with disability go online (Zickuhr and Smith 2012). About 100 million Americans do not have broadband access, which limits the speed of Internet access (FCC 2012). About 50% of all Americans own a smartphone, but only 13% of seniors aged 65 and older (Smith 2012). People who are older, disabled, poorer, or less educated are less likely to have Internet access or a smartphone. This is of concern since many patients with bipolar disorder are unemployed or disabled (Marwaha et al. 2013; Drake et al. 2013). Currently, paper handouts are still recommended for information that should be read by all patients with bipolar disorder.

Another aspect of the digital divide relates to the knowledge necessary to successfully search for health information on the Internet. Many people lack Internet skills such as how to use a search engine or save a PDF file, especially seniors or those with lower levels of education regardless of age (van Deursen 2012). Self-reported computer literacy may be unreliable (Merritti et al. 2005), and even members of the so-called ‘net generation’ are not universally comfortable or savvy about the web. For example, considerable variation was found in the skills needed for seeking information online among a freshman class of 1,600 at a public university (Hargittai 2010). In addition to technical skills, the language gap between laypersons and professionals regarding medical concepts makes it difficult to search and appraise content (Zeng and Tse 2006). Frequent use of slang, abbreviations, and spelling errors in medical terminology by laypersons all contribute to unsuccessful searches (McCray et al. 1999). The language problem is exacerbated by the use of general search engines. For example, using ‘the blues’ as a search term in Google resulted in all first page results related to music. Using ‘the blues’ as the search term in the specialized MedLinePlus search engine, resulted in 4 of 10 first page results related to depression. Furthermore, many people have complex medical questions, and answers are not directly available on health web sites (McCray et al. 1999). Since patients with bipolar disorder frequently take polypharmacy (Baldessarini et al. 2008; Bauer et al. 2013a) and have medical comorbidity (McIntyre et al. 2006), many unique queries would be expected suggesting a need for advanced searching skills. Finally, there are only limited Spanish language web pages in US health web sites (Berland et al. 2001).

### Encourage patients to seek health information on the internet

Despite the wide range of cautionary issues, the benefits remain and patients with bipolar disorder should be encouraged to search for health information on the Internet. Physicians should offer to discuss information that patients read on health web sites. There are many web sites related to mental health on the Internet, as a search of ‘bipolar disorder’ on Google returned 14,300,000 hits. Given the mixed content quality and uneven privacy policies of health web sites, physicians should assist patients in their search by providing a brief printed handout on concerns associated with Internet searching, along with recommending web sites they have reviewed as appropriate for their patients. Both psychiatric and general medical information web sites should be included. The content of US government sites, such as from the NIH, NIMH, CDC, and FDA, are evaluated for accuracy and currency (Miller et al. 2004). In addition to reliable content, health web sites from the government and professional organizations may be less likely to leak personal information (Huesch 2013). A physician may also want to recommend sites for uses other than information searching, such as to obtain financial assistance for brand name prescription drugs, or sites related to patient advocacy. Given the frequent disappearance of low traffic health web sites, a list with a small number of recommended sites may be appropriate. One study of healthcare management journals found that that 50% of URLs could not be located after 3 to 5 years (Wagner et al. 2009).

## Conclusion

Adults regularly use the Internet to obtain health information. Given the complexity of bipolar disorder, physicians should support patients with bipolar disorder and caregivers who want to search for information online, and offer to discuss what they read. The digital divide remains and includes technical skills and searching competence, as well as Internet access. Patients, caregivers, and physicians should be aware of potential concerns when searching for health information on the Internet, including privacy issues, content quality, and consumer fraud. To facilitate the search for health information on the Internet, physicians should provide patients with a list of trustworthy sites and a brief summary of concerns. More studies of the use of the Internet to search for health information by patients with bipolar disorder are needed.
